# Dopaminergic neuron metabolism: relevance for understanding Parkinson’s disease

**DOI:** 10.1007/s11306-024-02181-4

**Published:** 2024-10-13

**Authors:** Xóchitl Flores-Ponce, Iván Velasco

**Affiliations:** 1 Instituto de Fisiología Celular – Neurociencias, Universidad Nacional Autónoma de México, Mexico City, Mexico; 2 Laboratorio de Reprogramación Celular, Instituto Nacional de Neurología y Neurocirugía “Manuel Velasco Suárez”, Mexico City, Mexico

**Keywords:** Metabolic alterations, Neurodegeneration, Aging, Complex axonal arborization, Pacemaking activity, ROS production

## Abstract

**Background:**

Dopaminergic neurons from the *substantia nigra pars compacta* (SNc) have a higher susceptibility to aging-related degeneration, compared to midbrain dopaminergic cells present in the ventral tegmental area (VTA); the death of dopamine neurons in the SNc results in Parkinson´s disease (PD). In addition to increased loss by aging, dopaminergic neurons from the SNc are more prone to cell death when exposed to genetic or environmental factors, that either interfere with mitochondrial function, or cause an increase of oxidative stress. The oxidation of dopamine is a contributing source of reactive oxygen species (ROS), but this production is not enough to explain the differences in susceptibility to degeneration between SNc and VTA neurons.

**Aim of review:**

In this review we aim to highlight the intrinsic differences between SNc and VTA dopamine neurons, in terms of gene expression, calcium oscillations, bioenergetics, and ROS responses. Also, to describe the changes in the pentose phosphate pathway and the induction of apoptosis in SNc neurons during aging, as related to the development of PD.

**Key scientific concepts of review:**

Recent work showed that neurons from the SNc possess intrinsic characteristics that result in metabolic differences, related to their intricate morphology, that render them more susceptible to degeneration. In particular, these neurons have an elevated basal energy metabolism, that is required to fulfill the demands of the constant firing of action potentials, but at the same time, is associated to higher ROS production, compared to VTA cells. Finally, we discuss how mutations related to PD affect metabolic pathways, and the related mechanisms, as revealed by metabolomics.

## Introduction

Dopaminergic (DA) neurons located in the ventral midbrain are anatomically divided into different regions: *substantia nigra pars compacta* (SNc; group A9), ventral tegmental area (VTA; group A10), and retrorubral field (RRF; group A8) nuclei. DA neurons found in the SNc project mainly their axons to locomotor areas innervating the dorsolateral region of the caudate-putamen in a rostral, medial, and caudal gradient (nigrostriatal pathway) (Garritsen et al., 2023; Poulin et al., 2014). However, recent evidence showed that DA neurons located in the lateral and dorsolateral tiers of the SNc project to the prefrontal cortex (mesoprefrontal pathway), which is involved in the control of cognitive functions such as working memory, attention, and adaptability (Matsumoto & Takada, 2013; Ott & Nieder, 2019). On the other hand, dopamine neurons from the VTA display projections to the nucleus accumbens, olfactory tubercle, the amygdala (mesolimbic pathway), and to the prefrontal and entorhinal cortex (mesocortical pathway), playing important roles in motivation, reward and cognition (Björklund & Dunnett, 2007; Garritsen et al., 2023).

Impairments in the nigrostriatal pathway result in basal ganglia dysfunction and motor alterations, leading to the development of Parkinson’s disease (PD). PD is a neurodegenerative condition characterized by a prominent early loss of dopaminergic neurons in the SNc, which progresses over time (Fearnley & Lees, 1991; Hirsch et al., 1988; Kordower et al., 2013; Surmeier et al., 2017). The main clinical manifestations comprise motor symptoms such as bradykinesia, resting tremor, muscular rigidity, and postural instability in gait (Bloem et al., 2021; Kalia & Lang, 2015). Loss of SNc DA neurons has been associated with age, exposure to toxins, environmental factors, and genetic mutations (Poewe et al., 2017). Nevertheless, loss of dopaminergic neurons in the VTA has also been observed in PD, although this loss is more evident in advanced stages of the disease. Postmortem analyses of brain tissues from PD patients revealed that the number of DA neurons in the SNc significantly decreases (> 70%), followed by a less-severe loss of DA neurons in the VTA (∼50%) (Alberico et al., 2015; Damier et al., 1999; Hirsch et al., 1988; Surmeier et al., 2017). In line with these findings, the content of Tyrosine Hydroxylase (TH), a crucial protein in the synthesis of dopamine, decreased first in the SNc after injection of 6-hydroxydopamine (6-OHDA) in the rat brain; nonetheless, a significant reduction in TH levels was also observed in the VTA in advanced stages of the 6-OHDA lesion (Kasanga et al., 2023). In non-human primate models of the disease, DA neurons from the SNc are also more susceptible to toxins that induce oxidative stress, such as MPTP (1-methy-4-phenyl-1,2,3,6-tetrahydropyridine), that causes neurodegeneration by inducing mitochondria dysfunction (Chiueh et al., 1985; Dopeso-Reyes et al., 2014; Schneider et al., 1987). The preferential damage caused by this compound suggests that SNc neurons are more vulnerable to energy impairments and reactive oxygen species (ROS).

Previous studies also suggest that an increase in ROS after spontaneous oxidation of cytosolic dopamine could contribute to exacerbated dopaminergic neuron degeneration in PD (Asanuma et al., 2004). However, although dopamine represents an important source of free radicals and highly reactive metabolites, it is not sufficient to explain the differential vulnerability to degeneration observed between dopaminergic neurons from the SNc and the VTA, since both produce dopamine. Therefore, some intrinsic features might contribute to make one population more susceptible to damage. In support of this notion, recent studies have revealed that DA neurons in the SNc display important metabolic differences, compared to those DA neurons in the VTA, that might contribute to increase their vulnerability (Pacelli et al., 2015). Such intrinsic differences between SNc and VTA DA neurons include calcium oscillations, high ATP production, limited reserve respiratory capacity, and a high rate of mitochondrial DNA mutations induced by oxidative species.

In this review, we focus on the metabolism of DA neurons in the SNc, and how this metabolic signature contributes to their increased susceptibility for neurodegeneration when they are exposed to genetic and environmental factors, and to normal aging, comparing them with the closely related VTA DA neurons. Recent advances suggest that alterations in glucose and energy metabolism in these neurons could play an important role in the onset and progression of PD.

## Metabolic signature of dopaminergic neurons in the SNc

### Energy metabolism in SNc DA neurons

The brain is an organ with a high-demand metabolic rate. In resting state, the human brain requires about 20% of the calories available for the whole body (Rolfe & Brown, 1997). According to positron emission tomography studies, this is equivalent to a consumption rate of 5.9 mg of glucose per 100 g of brain tissue, per minute (Blomqvist et al., 1994; Reivich et al., 1979). This is an important consumption rate, considering its small size, relative to the total body weight (Rolfe & Brown, 1997). In order to meet high metabolic demands, the brain produces large amounts of energy, mainly from glucose oxidation (Raichle & Gusnard, 2002). However, each of the cell types present in the brain differs in glucose utilization. In particular, neurons required a large portion of energy to sustain cellular processes associated to signaling, such as propagation of action potentials and restoration of ion fluxes in postsynaptic terminals after neurotransmission stimulation (Bonvento & Bolaños, 2021; Harris et al., 2012). Nevertheless, different neuronal subtypes display a unique metabolic signature, according to its morphology and function.

Previous studies have shown that DA neurons from the SNc exhibit a large and highly dense axonal arborization compared to other midbrain dopaminergic neurons (Table 1) (Matsuda et al., 2009). According to morphological analysis, the total axonal length of a single DA neuron from the SNc reaches between 500,000 and 610,000 µM and exhibits a high number of axonal processes (Matsuda et al., 2009). These features are also maintained in cultured SNc DA neurons that show 2 and 3-fold greater axonal length and arborization, respectively, compared to VTA DA neurons (Pacelli et al., 2015). Given its complexity, it has been estimated that a single SNc neuron can innervate 2.7% of the total volume of the rat striatum and give rise to between 100,000 and 245,000 synaptic contacts. In humans, the number of synapses per SNc dopaminergic neuron increases tenfold, reaching between 1 and 2.4 million synapses (Bolam & Pissadaki, 2012; Matsuda et al., 2009).

**Table 1:**
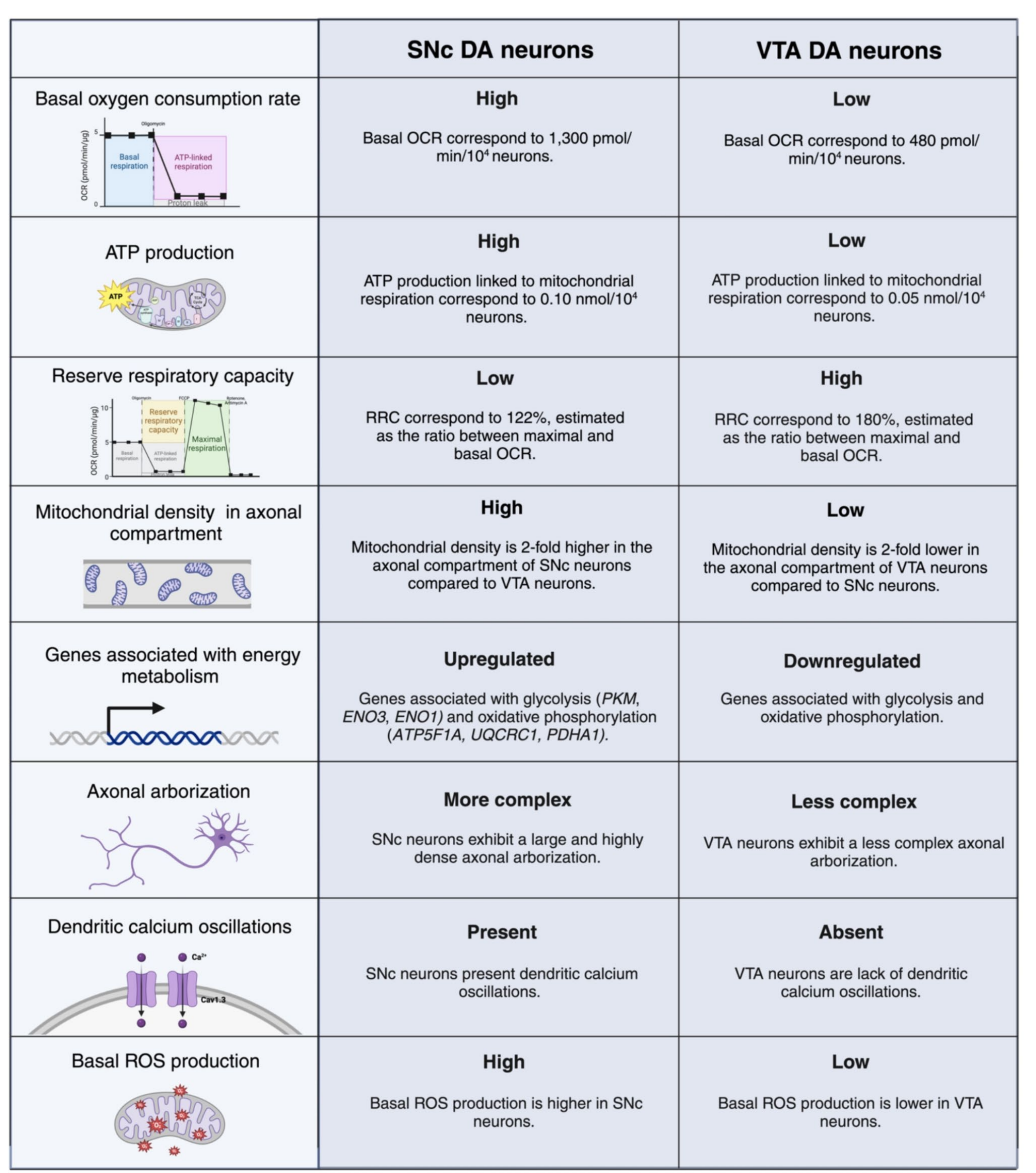
Comparison of processes associated with energy metabolism in SNc and VTA dopaminergic neurons

Unlikely most neurons in the CNS, midbrain dopaminergic neurons are able to generate action potentials (APs) at a rate of 2–4 Hz in the absence of electrical stimuli. This continuous firing, known as autonomous pacemaking, plays an important role in the optimal maintenance of motor functions through the constant release of dopamine in the striatum (Guzman et al., 2009). Studies have shown that SNc DA neurons robustly express L-type Cav1.3 calcium channels, which allow a prominent calcium influx during pacemaking activity (Table 1) (Bonci et al., 1998; Guzman et al., 2010; Puopolo et al., 2007), whereas VTA DA neurons use sodium channels to drive pacemaking (Chan et al., 2007). These calcium currents support autonomous pacemaking and also sustain an elevated spike rate after synaptic stimulation, facilitating the propagation of APs throughout the complex axonal terminals of SNc DA neurons (Pissadaki & Bolam, 2013; Zampese et al., 2022).

Intracellular calcium levels are regulated within SNc DA neurons by ATP-dependent mechanisms, including endoplasmic reticulum and mitochondrial calcium sequestering, and also calcium extrusion by membrane transporters (Foehring et al., 2009; Surmeier, 2007). In fact, calcium oscillations promote Ca^2+^ release from the endoplasmic reticulum, stimulating mitochondrial oxidative phosphorylation (OXPHOS) (Zampese et al., 2022). Since Ca^2+^ buffering is a high-energy consuming process, SNc DA neurons require elevated ATP production, leading to an increase in reactive oxygen species (ROS) levels (Pissadaki & Bolam, 2013). Further, studies have shown that SNc DA neurons exhibit higher levels of mitochondrial matrix oxidant stress, as a consequence of calcium influx, compared to VTA DA neurons (Guzman et al., 2010).

Current findings show that SNc neurons display a higher mitochondrial energy metabolism compared to other midbrain dopaminergic neurons, as a result of its large and complex axonal arborization (Pacelli et al., 2015). In basal conditions, SNc neurons show a high oxygen consumption rate (OCR) and elevated ATP production compared to VTA neurons, implying that ATP production mainly derives from oxidative phosphorylation (OXPHOS) rather than glycolysis (Table 1) (Pacelli et al., 2015). Consistent with these findings, computational approaches have shown that neurons with a complex axonal architecture require a higher basal energy production than less complex neurons, in order to sustain neurotransmission (Pissadaki & Bolam, 2013).

Usually, cells are able to generate extra amounts of ATP through OXPHOS in response to unexpected increases in energy demands. Interestingly, this reserve respiratory capacity (RRC) is lower in SNc neurons compared to VTA neurons, suggesting that SNc neurons are less capable of meeting additional energy demands than VTA neurons, because they operate close to their highest energy production capacity in basal conditions (Table 1) (Pacelli et al., 2015).

In addition to elevated ATP production and high OXPHOS rate, SNc neurons show a larger mitochondrial density compared to VTA neurons (Table 1). These are consistent with the increased expression of the transcriptional coactivator peroxisome proliferator-activated receptor-coactivator 1α (PGC-1α) observed in SNc neurons (Pacelli et al., 2015). PGC-1α is a master regulator that induce the expression of a set of genes that are crucial for mitochondrial function, energy metabolism, and biogenesis (Rohas et al., 2007). Moreover, genes implicated in energy metabolism are overexpressed in SNc neurons, such as ATP Synthase F1 Subunit Alpha (*ATP5F1A*), Ubiquinol-Cytochrome C Reductase Core Protein 1 (*UQCRC1*), and Dehydrogenase E1 Subunit Alpha 1 (*PDHA1*) that participated in OXPHOS and Pyruvate Kinase (*PKM*), Enolase 3 (*ENO3*), and Enolase 1 (*ENO1*) crucial in glycolysis (Table 1) (Greene et al., 2005).

#### Oxidative stress as a consequence of a high energy metabolism

In VTA neurons, the basal mitochondrial oxidative stress is very low, compared to SNc neurons. In agreement, SNc neurons display significantly higher levels of basal production of mitochondrial ROS (Table 1). Different from the effect on pacemaking activity, antagonizing L-type channels significantly diminished the basal ROS production in SNc DA neurons. This indicates that influx of Ca^2+^ through L-type calcium channels during autonomous pacemaking increases basal mitochondrial oxidative stress in SNc DA neurons (Guzman et al., 2010). On the other hand, it is well known that SNc DA neurons are more vulnerable to toxins that cause oxidative stress, such as MPTP, compared to VTA DA neurons. Interestingly, inhibition of calcium channels by isradipine significantly reduces their vulnerability and protects SNc DA neurons from cell death after MPP^+^ exposition. Furthermore, inhibition of calcium channels substantially reduces mitochondrial respiration, reflected as a decrease in basal OCR (Pacelli et al., 2015).

### Glucose metabolism via the pentose phosphate pathway in SNc DA neurons

Neurons maintain their ATP production mainly through oxidative phosphorylation, whereas astrocytes use glycolysis. When mitochondrial respiration is disrupted, neurons and astrocytes respond differently at the metabolic level. Astrocytes are able to increase the rate of glycolysis after inhibiting mitochondrial respiration by nitric oxide, potassium cyanide, or oligomycin. However, this glycolytic response does not occur in neurons, even though cellular respiration is inhibited. The disruption in the respiratory chain is followed by a progressive decrease in ATP levels, mitochondrial depolarization, and cell death in neurons (Almeida et al., 2001).

**Fig. 1:**
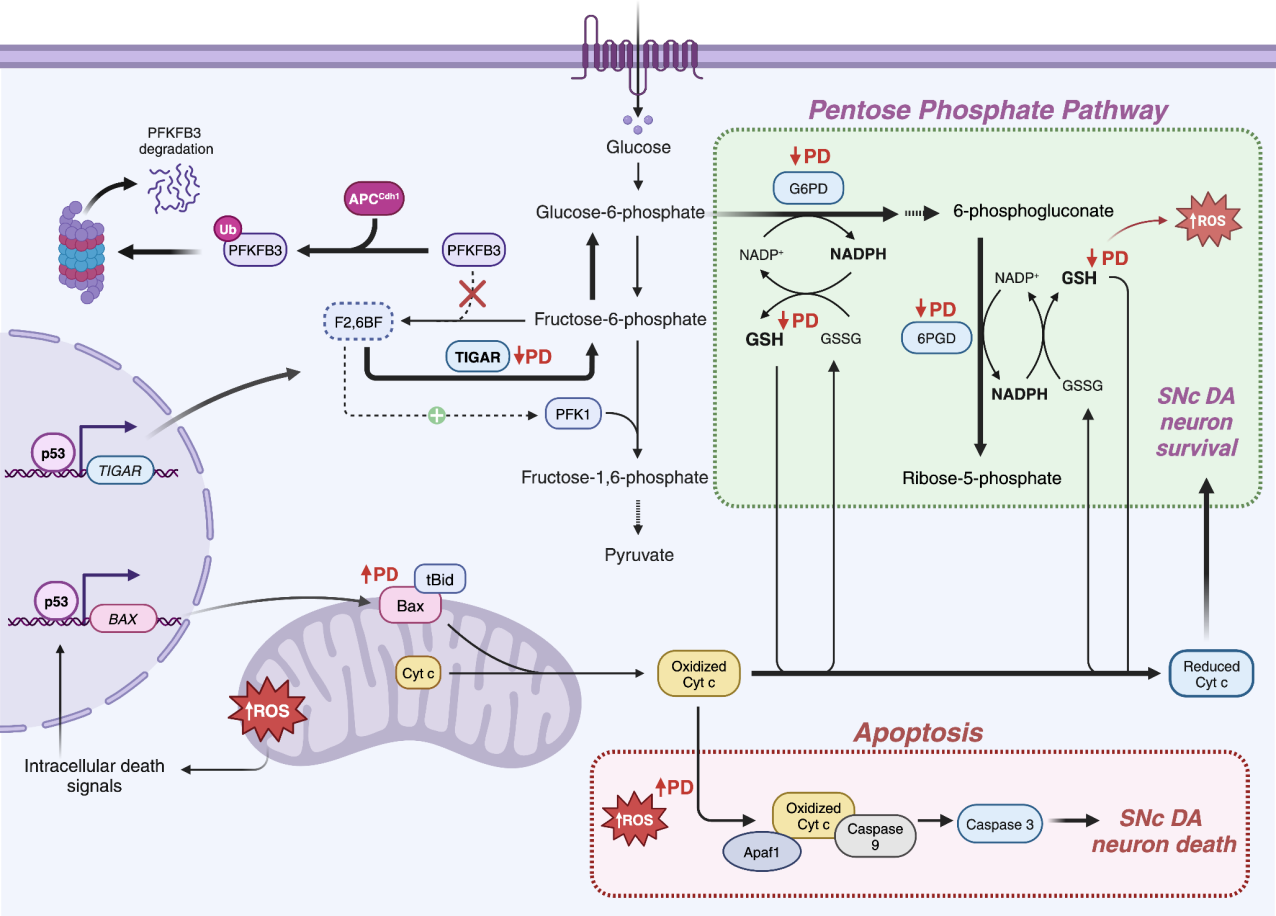
Dysregulation of pentose phosphate pathway in Parkinson’s disease. In healthy DA neurons, the glycolytic flux is regulated by PFKFB3 and F2,6BF levels. The proteosome degradation of PFKFB3 decreases the concentrations of F2,6BF, a crucial activator of PFK1, thus, reducing glycolytic pathway, and favoring the PPP, which allows production of antioxidant molecules such as glutathione (GSH). p53-mediated regulation of TIGAR enhance the conversion of F2,6BF to fructose-6-phosphate, increasing the flux to PPP. In early-stages of PD, enzymes that are essential in this pathway such as glucose-6-phosphate dehydrogenase (G6PD), and 6-phosphogluconate dehydrogenase (6PGD), are downregulated, resulting in decreased levels of GSH. Increases in mitochondrial ROS levels trigger intrinsic apoptotic signaling. In PD, TIGAR levels are decreased, because this protein is included in Lewy bodies

The upregulation of glycolysis observed in astrocytes after inhibition of mitochondrial complexes relies on the phosphorylation of the AMPK in response of increased ADP/ATP ratios. Once activated, AMPK increased the activities of PFKFB3 (6-phosphofructo-2-kinase/fructose-2,6-biphosphatase 3). This glycolytic enzyme catalyzes the synthesis and degradation of fructose-2,6-biphosphate (F2,6BP), a master regulator of glycolysis. F2,6BP is crucial for the activation of PFK1 (phosphofructokinase 1) in the glycolytic pathway. Thus, the levels of F2,6BP determine the glycolytic rate in cells (Almeida et al., 2004). Interestingly, in neurons, PFKFB3 is downregulated post-translationally. This glycolytic enzyme is degraded through the ubiquitin-proteosome pathway by the action of the E3 ubiquitin ligase APC/C-Cdh1 (Fig. 1). Whereas, in astrocytes, PFKFB3 is stable through the downregulation of this ubiquitin ligase (Herrero-Mendez et al., 2009). Therefore, neurons actively downregulate glycolysis, and preferentially metabolize glucose through the pentose phosphate pathway (PPP), in contrast to other cell types. This pathway is critical to maintain the key molecule antioxidant glutathione (GSH) in a reduced state, regulating oxidative stress levels (Fig. 1). The upregulation of glycolysis results in a significant decrease in the glucose flux through the PPP, leading to an increase in ROS levels and neuronal death (Herrero-Mendez et al., 2009).

Increased ROS levels can trigger intracellular death signals through activation of the tumor suppressor protein, p53 (Fig. 1) (Bensaad et al., 2006). This results in the release of cytochrome c from the mitochondria to the cytoplasm. Interestingly, neurons inhibit cytochrome c-mediated apoptosis through direct inactivation of oxidized cytochrome c, which is reduced and maintained inactive by GSH, leading to long-term neuronal survival (Fig. 1) (Vaughn & Deshmukh, 2008). Additionally, APC/C-Cdh1 prevents the accumulation of cyclin B1 in terminally differentiated neurons, playing a key role in the survival of postmitotic neurons (Almeida et al., 2005).

As described above, dopaminergic neurons from the SNc are exposed to high levels of oxidative stress as a response to a high OXPHOS rate. For this reason, optimal maintenance of the PPP is essential to sustain the antioxidant glutathione system, important to decrease intracellular ROS levels in SNc neurons. Recent studies have shown that the PPP is altered in the putamen and cerebellum in the early stages of PD. There is a reduction in glucose-6-phosphate dehydrogenase (G6PD) and 6-phosphogluconate dehydrogenase (6PGD) levels, two enzymes that are crucial in the PPP (Fig. 1) (Dunn et al., 2014). Moreover, levels of reduced glutathione (GSH) are significantly lower in the SNc (∼40%) of sporadic PD patients (Sian et al., 1994; Sofic et al., 1992). In addition, TIGAR (TP53-Induced Glycolysis and Apoptosis Regulator), a protein that regulates ROS levels through the PPP, has been found in Lewy bodies in the substantia nigra of sporadic PD patients (Fig. 1) (Bensaad et al., 2006; López et al., 2019). In contrast, glutathione peroxidase 1 (GPX1), an enzyme that participates together with GSH to decrease ROS, is expressed at higher levels in DA neurons of the VTA compared to nigral DA neurons in PD patients (Smeyne & Smeyne, 2013). On the other hand, the expression levels of hexokinase 2 (HK2) and lactate dehydrogenase A (LDH-A) were increased in SNc DA neurons in the brain of MPTP-injected mouse model of PD, and also in MPP^+^-treated catecholaminergic SH-SY5Y cells (Li et al., 2022b). High levels of HK2 and LDH-A produce an increase in the glycolytic rate (Chen et al., 2016), which results in the accumulation of lactate and the triggering of apoptosis in SNc neurons through the AMPK/Akt/mTOR pathway (Li et al., 2022b).

## Energy metabolism alterations during normal aging in SNc DA neurons

### Mitochondrial DNA deletions in SNc DA neurons during aging

Several studies have revealed that DA neurons of the SNc are more prone to accumulate mitochondrial DNA (mtDNA) deletions during aging. SNc DA neurons show high levels of mtDNA deletions (33.9–60%) compared to VTA DA neurons (21.9%) and neurons from distinct brain regions, such as the hippocampus, the frontal cortex, and the putamen (∼14%) (Bender et al., 2006, 2008; Elstner et al., 2011; Kraytsberg et al., 2006). Moreover, DA neurons with high levels of mtDNA deletions show a decrease in complex IV (Cytochrome c oxidase) activity. This mtDNA-encoded enzyme plays a crucial role in mitochondrial respiration, suggesting that these mutations may be associated with mitochondrial respiratory chain dysfunction (Bender et al., 2006, 2008; Itoh et al., 1996; Kraytsberg et al., 2006). It seems most likely that elevated levels of ROS from a high-energy metabolism may induce mtDNA deletions in SNc DA neurons. Therefore, the disruption of mitochondrial respiratory chain function, associated with mtDNA deletions, could trigger an early axonal degeneration of SNc dopaminergic neurons.

### Metabolic changes in SNc DA neurons during aging

Several studies reveal that the brain undergoes important metabolic changes during aging (Cleeland et al., 2019). In particular, whole brain magnetic resonance spectroscopy shows that the midbrain of elderly people displays differences in N-acetyl-L-aspartate (NAA) and choline-containing compounds (Cho) levels. (Moreno-Torres et al., 2005). The concentration of the metabolite NAA significantly decreases in the midbrain, tegmentum, and putamen at a rate of 3.8% and 4.9% per decade, respectively (Maghsudi et al., 2020). Evidence suggests that a reduction in NAA levels is associated with neuronal dysfunction and loss (Stefano et al., 1995). Intriguingly, NAA decreases during aging more rapidly in the midbrain and putamen than in other brain areas, indicating a reduction in neuronal density and function (Maghsudi et al., 2020).

### Loss of SNc dopaminergic neurons in normal aging

Morphometry studies have shown a decrease of 33% in the total count of pigmented neurons of the SNc between 20- and 90-years-old control cases. That means a rate of 4.7% loss per decade due to aging (Fearnley & Lees, 1991). Additional studies found a similar decrease in the number of dopaminergic neurons in SNc, with a loss of 4.7–6.0% per decade from 50 to 90 years of age (Gibb & Lees, 1991). These findings are consistent with the reduction of dopamine levels observed in the caudate (54 to 68%) and putamen (46 to 68%) nuclei in postmortem brains of healthy aged humans (Kish et al., 1992). These studies did not report a VTA DA neuron decline associated to aging in humans. Non-human primates also exhibit an age-related decline in SNc DA neurons. However, the number of dopaminergic neurons in the VTA does not decrease significantly with aging in monkeys (Emborg et al., 1998; Kanaan et al., 2007). In other mammals, such as rodents, it has been reported that the SNc of aged rats shows an important decrease in TH protein content, accompanied by a significant reduction in dopamine levels. In these aged rats, there are no significant changes in TH, nor in dopamine levels, in the VTA (Salvatore et al., 2009).

It is possible that SNc DA neuron loss could explain the motor decline observed in humans and non-human primates at advanced age (Sohmiya et al., 2001). This is relevant considering that aging is the major risk factor for developing PD. Even though this neuronal loss observed during normal aging is not enough to cause PD, the factors that promote its loss could contribute to neurons being more vulnerable to neurodegeneration when exposed to genetic and environmental factors.

## Metabolic dysfunction in Parkinson’s disease

### Metabolic alterations in sporadic PD

Sporadic PD has been associated with mitochondrial complex I dysfunction (Schapira et al., 1990). According to this, several studies have revealed that there is an increase in the glycolytic rate and lactate levels in sporadic PD patients (Bowen et al., 1995; Hu et al., 2000). Furthermore, Meta-Gene Set Enrichment Analysis (GSEA) of postmortem brain tissue of patients with idiopathic PD has shown evidence of energy-producing dysfunction in nigral DA neurons: a metabolic switch to aerobic glycolysis. Meta-analysis identified 10 molecular pathways comprising gene sets related to a biological process associated with altered energy and glucose metabolism, in early and late stages of PD. The electron transport chain (ETC), OXPHOS, PGC-1α, pyruvate metabolism, tricarboxylic acid (TCA) cycle, and carbohydrate-responsive element–binding protein (ChREBP) gene sets were downregulated in PD. Interestingly, ChREBP is a transcription factor that transactivates key genes of glucose metabolism (Zheng et al., 2010). Metabolic alterations specifically for the VTA have not been reported, and it will be interesting to investigate if there is a relationship of metabolic dysfunction with the late degeneration of VTA neurons observed in PD.

These metabolic changes indicated that impairments in energy metabolism are enough to trigger, in SNc DA neurons, a metabolic reprogramming from OXPHOS to aerobic glycolysis. Evidence indicates that the induction of a metabolic switch toward glycolysis increases the susceptibility to apoptotic stimuli in healthy neurons (González-Rodríguez et al., 2021).

### Metabolic alterations linked to mutations associated with familial PD

Several genes that have been associated with familial forms of PD play an important role in the metabolism of glucose. Particularly, mutations in *Park2*, *Pink1* and *Park7* promote an altered state of glucose and energy metabolism that could be implicated in the onset and progression of PD.

#### PARK2

**Fig. 2:**
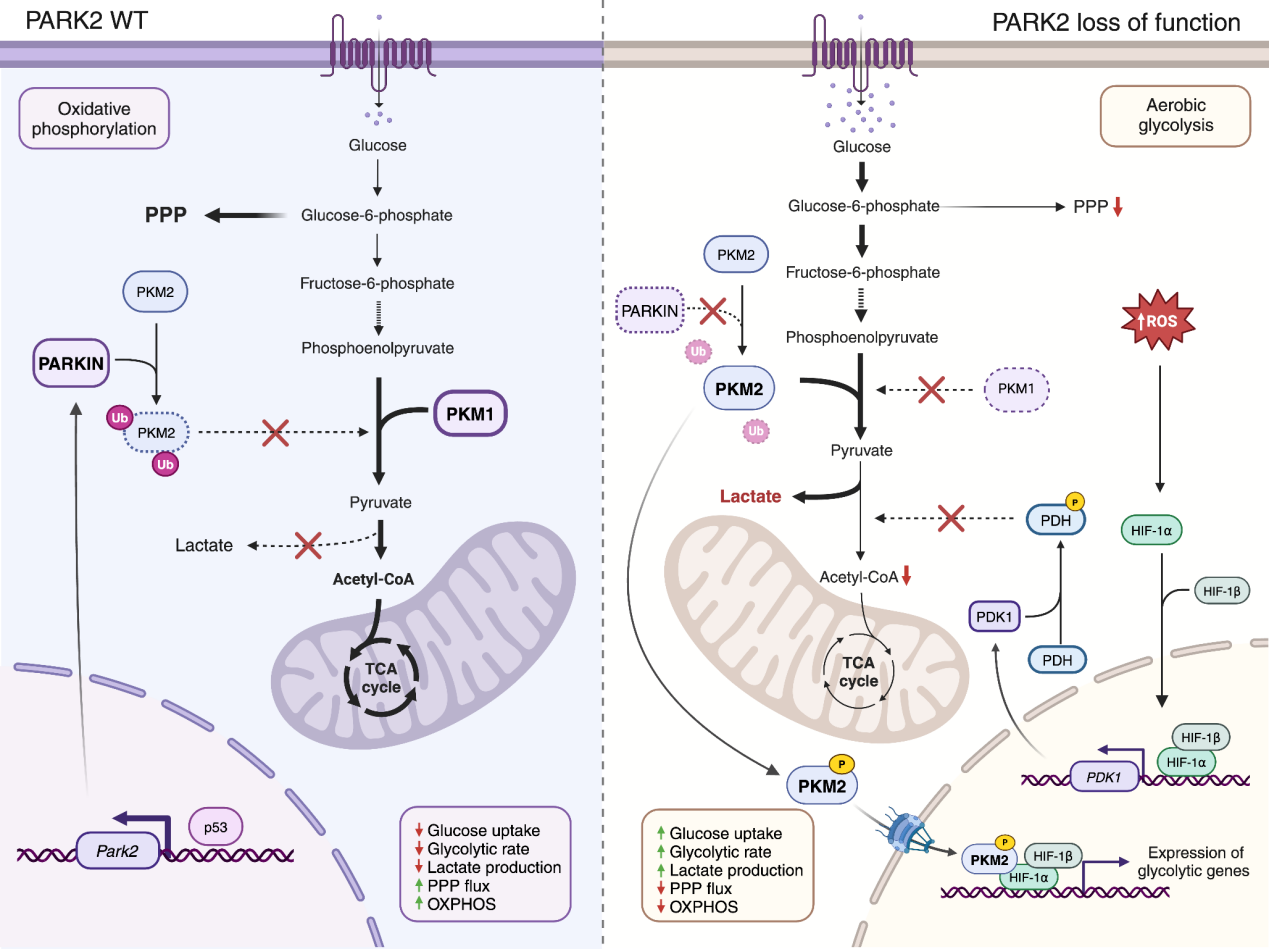
Metabolic alterations linked to mutations in *Park2*. In normal conditions, Parkin *(Park2)* decreases the enzymatic activity of pyruvate kinase 2 (PKM2) through ubiquitination, thus regulating energy metabolism and antioxidant defense through p53. Loss of function of Parkin results in an enhancement of PKM2 activity and a downregulation of pyruvate kinase 1 (PKM1), leading to an upregulation of the glycolytic rate. Phosphorylated PKM2 translocates to the nucleus and supports the expression of glycolytic genes through hypoxia-inducible factor-1α (HIF-1α). Additionally, Parkin deficiency increases ROS levels, that induce pyruvate dehydrogenase kinase-1 (PDK1) upregulation, through HIF-1α stabilization, resulting in a reduction of pyruvate dehydrogenase (PDH) activity. This leads to a reduction of acetyl-CoA levels and mitochondrial respiration, promoting an enhanced glucose metabolism. Thus, the loss of Parkin triggers a metabolic shift toward aerobic glycolysis. PPP: pentose phosphate pathway

Mutations in Parkin (*PARK2*), a ubiquitin E3 ligase, have been associated with recessive forms of juvenile PD (Kitada et al., 1998). Studies have shown that Parkin plays an important role in regulating energy metabolism and the antioxidant defense through p53. The protein p53 binds to a responsive element located in PARK2, increasing Parkin transcription (Fig. 2) (Zhang et al., 2011). p53 is a transcription factor that induces, in response to stress, the expression of genes that activate cellular mechanisms associated with cell cycle arrest, apoptosis, and senescence in order to suppress tumor formation (Matoba et al., 2006). However, studies reveal that p53 also plays a key role in energy metabolism, regulating glycolysis and oxidative phosphorylation to sustain energy demands and regulating intracellular ROS levels (Bensaad et al., 2006; Matoba et al., 2006).

In glycolysis, pyruvate kinase (PKM1) catalyzes the conversion of phosphoenolpyruvate to pyruvate, a crucial step in this pathway. On the other hand, pyruvate kinase M2 (PKM2) is a less active isoform of pyruvate kinase that is expressed during embryonic development and is upregulated in tumor tissues (Christofk et al., 2008). Studies have shown that Parkin decreases the enzymatic activity of pyruvate kinase 2 (PKM2) through ubiquitination (Fig. 2) (Liu et al., 2016). Loss of function of Parkin results in an enhancement of PKM2 activity and a downregulation of PKM1, leading to an upregulation of the glycolytic rate (Fig. 2) (Liu et al., 2016). Moreover, the phosphorylation of PKM2 leads to its nuclear translocation and supports the expression of glycolytic genes through hypoxia-inducible factor-1α (HIF-1α) (Luo et al., 2011).

Mass spectrometry analyses have revealed that the loss of function of Parkin increases levels of glucose 6-phosphate, fructose 6-phosphate, 3-phosphoglycerate, phosphoenolpyruvate, and pyruvate (Liu et al., 2016). Additionally, Parkin deficiency decreases the levels and activity of pyruvate dehydrogenase (PDH), which results in a reduction of acetyl-CoA and mitochondrial respiration, promoting an enhanced glucose metabolism (Fig. 2) (Zhang et al., 2011). This contributes to a decrease of GSH levels and, thus, an increase in ROS levels (Palacino et al., 2004). In summary, Parkin deficiency results in alterations of glycolysis, triggering in cells a metabolic shift toward aerobic glycolysis.

#### PINK1

Mutations in PTEN-induced kinase-1 (*PINK1)* gene have been associated with an early-onset form of PD (Valente et al., 2004). Studies have revealed that neurons carrying *PINK1* mutations are more susceptible to cellular stress and apoptotic cell death (Valente et al., 2004). Moreover, loss of function of PINK1, disrupts mitochondrial autophagy and contributes to an increase in ROS levels (Requejo-Aguilar et al., 2014). The elevated ROS levels promote the stabilization of hypoxia-inducible factor-1α (HIF-1α). This factor is a master transcriptional regulator that induces the expression of glycolytic genes in response to hypoxia (Weidemann & Johnson, 2008). The upregulation of HIF-1α enhances the expression of PDK1, resulting in the inhibition of PDH activity (Requejo-Aguilar et al., 2014). Furthermore, loss of Pink1 causes the upregulation in mRNA and protein levels of main glycolytic enzymes, including glucose transporter-1 (*Glut1*), hexokinase-2 (*Hk2*), and glyceraldehyde-3 phosphate dehydrogenase (*Gapdh*) (Requejo-Aguilar et al., 2014). Thus, Pink1-deficient neurons show a significant increase in glucose uptake and lactate production, and a decrease in the glucose flux via the PPP. The elevated glycolytic flux and the decrease in pentose-phosphate flux suggest that Pink1-deficient neurons suffer a glucose metabolism reprogramming from mitochondrial oxidative to glycolytic metabolism compromising the PPP (Requejo-Aguilar et al., 2014). Interestingly, the upregulation of *Pink1* represses glycolysis and cell proliferation (Requejo-Aguilar et al., 2015). These findings strongly suggest that PINK1 participates in the maintenance of energy metabolism and glucose metabolism through the PPP.

#### PARK7

Mutations in PARK7 have been associated with autosomal recessive forms of PD (Bonifati et al., 2003). Recent findings have shown that loss of function of PARK7 triggers the formation of glycerate-adducts of metabolites and proteins. Particularly, there is an accumulation of glycerate-adducts of glutamate, GSH, oxidized glutathione, glutamine, glycerophosphorylethanolamine (GPE), and lysine (Heremans et al., 2022). Moreover, it was observed that the loss of PARK7 promotes an increase in the highly reactive intermediate, cyclic-1,3-phosphoglycerate. In glycolysis, the metabolite 1,3-bisphosphoglycerate (1,3-BPG) suffers an intra-molecular attack and forms the intermediate metabolite cyclic-1,3-phosphoglycerate. This intermediate is highly reactive with the free amino groups present in metabolites and proteins, forming glycerate-adducts (Heremans et al., 2022). These findings reveal that PARK7 neutralizes the reactive intermediate and prevents the formation of glycerate-modified metabolites and proteins.

In addition, it has been reported that the downregulation of the glycolytic enzyme PGK-1 promotes the accumulation of 1,3-BPG, and is sufficient to generate glycerate-adducts, even if PARK7 is fully functional (Heremans et al., 2022). Intriguingly, patients with mutations in PGK-1 present nigrostriatal dysfunction and develop juvenile parkinsonism (Morales-Briceño et al., 2019). The neurodegeneration observed in these patients has been associated with energy metabolism impairments. However, consistent with current findings, probably the accumulation of 1,3-BPG in patients with PGK1 deficiency might be a key contributor to promote the increased of the highly reactive intermediate metabolite cyclic 1,3-phosphoglycerate, contributing to exacerbated DA neuron cell death in PD. Additionally, PARK7 deficiency in *Drosophila* and human cells increases the activity of glycolytic enzymes (Solana-Manrique et al., 2022).

## Metabolomics in Parkinson’s disease

Metabolomics is an emerging field that studies changes in biological systems by analyzing a high number of low-weight (< 1,500 Da), known as metabolites (Kaddurah-Daouk & Krishnan, 2009; Wishart, 2016). Since metabolites are the final product of the interaction between proteins encoded by the genome, and are also influenced by environmental factors, metabolomic approaches have a great potential for studying multifactorial diseases, such as PD, where both endogenous and exogenous factors might be involved in its onset and in its progression (Shao & Le, 2019; Zhang et al., 2021). In PD research, metabolomics has been widely used to identify potential biomarkers for early disease detection. Most of the studies have analyzed different biofluids such as blood, cerebrospinal fluid, and urine from healthy people and from PD patients (Ibáñez et al., 2015; Lei & Powers, 2013; X. Li et al., 2022a). Recently, there is a growing interest in using metabolomics in cellular and animal models of PD, to obtain novel information about metabolic changes that might result in a better understanding of the mechanisms behind dopaminergic neurodegeneration in PD.

The description of induced pluripotent stem cell (iPSC) obtained from reprogramming human somatic cells to a pluripotent state (Takahashi et al., 2007) allowed to obtain PD patients-derived iPSC (Soldner et al., 2009), which can be induced to produce midbrain DA neurons in culture. Metabolomics studies using iPSC-derived DA neurons from PD patients with *Parkin* mutations showed that these neurons exhibit high levels of citrate, succinate, malate, glutamate, and lactate, but reduced levels of glucose, pyruvate, and GSH/GSSG ratio, strongly suggesting alterations in the TCA cycle, glucose, and glutathione metabolism (Okarmus et al., 2021). Furthermore, a significant decrease in the content of both essential and non-essential amino acids has been described in iPSC-derived DA neurons (Peng et al., 2023). The in vitro differentiation of iPSC allows the formation of mesencephalic neural precursors, which will later progress to terminally differentiated DA neurons. Such precursors display increased amino acid levels, but similar alterations to those found in DA neurons in the glutathione antioxidant system; on the other hand, lipids such as sphingolipids and glycerophospholipids are increased in neural precursors (Cukier et al., 2022). Interestingly, neural precursor cells derived from sporadic PD iPSC also showed metabolic defects in the TCA cycle, yielding decreased concentrations of citrate, succinate and malate, which are correlated to lower levels of NADH (Schmidt et al., 2023). In animal models of PD, consisting of the injection of dopaminergic toxins such as 6-hydroxydopamine or MPTP to rodents, metabolomics experiments found changes in several metabolites related to energy metabolism and TCA cycle in the striatum; notably, there were increases of succinate, glutamate, creatine, taurine and GABA (Gao et al., 2013; Lu et al., 2018).

## Conclusions

The direct comparison between VTA and SNc DA neurons presented here demonstrated important differences: SNc have a more extensive axonal arborization, calcium oscillations, and pacemaker activity that results in higher basal metabolic demands, and enhanced ROS generation. In these neurons, there is an elevated ATP production, high mitochondrial density in axons, and an up-regulation in the expression of genes related to mitochondrial oxidative metabolism. The high metabolic demands in SNc DA neurons leave them with limited reserve respiratory capacity to respond to unexpected increases in energy requirements.

During aging, DA neurons from the SNc present respiratory chain dysfunctions, which are caused by ROS-induced deletions in mitochondrial DNA, a phenomenon also present in VTA DA neurons, albeit at lower frequency. People reaching 50 years of age have a low but constant rate of DA neuron loss in the SNc; however, this aging-related decrease is not always enough to develop PD. In this disease, apoptotic degeneration of SNc DA neurons might be caused by the shift in energy metabolism from mitochondrial oxidative to glycolysis; this enhanced glycolytic state decreases the PPP flux, which is necessary for antioxidants production.

The overall high energy requirements of SNc DA neurons decrease their capacity to handle external stimuli and genetic alterations, which make them more prone to degeneration. Since DA neuron loss is present in aged people, additional factors are required for the appearance of the characteristic symptoms of PD, and these challenges are most likely related to energy-related pathways. Metabolomics can help to elucidate some of the cellular and molecular alterations present both in neural precursors and in DA neurons, produced from PD-derived iPSC.

## Data Availability

No datasets were generated or analysed during the current study.
